# Deaths of despair: cause-specific mortality and socioeconomic inequalities in cause-specific mortality among young men in Scotland

**DOI:** 10.1186/s12939-020-01329-7

**Published:** 2020-12-04

**Authors:** Mirjam Allik, Denise Brown, Ruth Dundas, Alastair H. Leyland

**Affiliations:** MRC/CSO Social and Public Health Sciences Unit, University of Glasgow, Berkeley Square, 99 Berkeley Street, Glasgow, G3 7HR UK

**Keywords:** Deprivation, Inequalities in health, Mortality, Young men, Deaths of despair, Drug related deaths

## Abstract

**Background:**

Increasing mortality among men from drugs, alcohol and suicides is a growing public health concern in many countries. Collectively known as “deaths of despair”, they are seen to stem from unprecedented economic pressures and a breakdown in social support structures.

**Methods:**

We use high-quality population wide Scottish data to calculate directly age-standardized mortality rates for men aged 15–44 between 1980 and 2018 for 15 leading causes of mortality. Absolute and relative inequalities in mortality by cause are calculated using small-area deprivation and the slope and relative indices of inequality (SII and RII_L_) for the years 2001–2018.

**Results:**

Since 1980 there have been only small reductions in mortality among men aged 15–44 in Scotland. In that period drug-related deaths have increased from 1.2 (95% CI 0.7–1.4) to 44.9 (95% CI 42.5–47.4) deaths per 100,000 and are now the leading cause of mortality. Between 2001 and 2018 there have been small reductions in absolute but not in relative inequalities in all-cause mortality. However, absolute inequalities in mortality from drugs have doubled from SII = 66.6 (95% CI 61.5–70.9) in 2001–2003 to SII = 120.0 (95% CI 113.3–126.8) in 2016–2018. Drugs are the main contributor to inequalities in mortality, and together with alcohol harm and suicides make up 65% of absolute inequalities in mortality.

**Conclusions:**

Contrary to the substantial reductions in mortality across all ages in the past decades, deaths among young men are increasing from preventable causes. Attempts to reduce external causes of mortality have focused on a single cause of death and not been effective in reducing mortality or inequalities in mortality from external causes in the long-run. To reduce deaths of despair, action should be taken to address social determinants of health and reduce socioeconomic inequalities.

**Supplementary Information:**

The online version contains supplementary material available at 10.1186/s12939-020-01329-7.

## Introduction

The rise in mortality and burden of disease in high income countries among young people, particularly men, has received growing international attention [1, 2]. This coincides with findings of increasing rates of suicide, and drug and alcohol deaths among young and middle-aged men in many countries [3, 4], and in the US particularly mortality rates from these causes have been covered widely [5–7]. Some researchers have labelled deaths from alcohol, drugs and suicide collectively as “deaths of despair”, stemming from the protracted and cumulative effects of deteriorating labour market opportunities, weakening of traditional social structures (marriages, churches, labour unions) and, fundamentally, the loss of things that give life a meaning [6].

In the US, deaths of despair have also been linked to educational attainment, with rising mortality among those without a college degree and falling for those with a college degree, leading to increased absolute inequalities in mortality [6]. In Europe, absolute inequalities in all-cause premature mortality have generally reduced, but relative inequalities have increased in many countries [8, 9]. Evidence on specific causes and age groups is more limited, but increasing absolute inequalities in alcohol related mortality in several Eastern and Northern European countries have been highlighted [10]. In New Zealand, inequalities in mortality, particularly from suicides and unintentional injury, have widened among young adults (25–44 years) [11].

The increase in deaths of despair in US was initially seen as atypical among the developed nations [6], but growing mortality from these causes and among younger age groups in England has led to heightened attention to this problem also in the UK [12–14]. The concerns are particularly relevant for Scotland, where mortality rates have been high relative to the rest of the UK and Europe for decades [15, 16]. Compared to England and Wales, mortality rates in Scotland are especially high among young adults (aged 15–44) and from causes such as drug- and alcohol related poisonings, and self-harm [15, 17, 18]. While mortality from many causes (e.g. heart disease, cancer, stroke) has fallen substantially since the 1980s in Scotland, deaths from alcohol, suicides and particularly drugs have significantly risen [19], and the country now has the highest rate of drug deaths in Europe [20]. Similarly to the US, the increases in deaths of despair in Scotland have been linked to socioeconomic status (SES), with much higher increases in deprived areas [19].

While high mortality rates for men aged 15–44 in Scotland have been noted before [18], no study has specifically focused on studying long-term trends in mortality and in socioeconomic inequalities in mortality by different causes among this group of young men. We have focused on men as in Scotland mortality and inequalities in mortality from drugs, alcohol and suicides are multiple times higher among men than women [19]. Given the severity of the problem in the UK, US and potentially in other developed nations, such as New Zealand [11], a detailed focus on this demographic group is warranted. Although deaths in younger age groups contribute a relatively small proportion to all deaths (5% in 2018 in Scotland), they represent a high number of potential years of life lost and thereby also a high economic cost to the society [21, 22]. Our study makes a major contribution to this acute public health concern, by using high-quality national population-wide data, adding a longitudinal dimension, and looking at causes of death specific to younger ages, including the deaths of despair.

## Data and methods

We analyse trends in mortality across four decades from 1980 to 2018 and trends in socioeconomic inequalities in mortality between 2001 and 2018, the latter period defined by the availability of small-area deprivation data. The data on deaths and mid-year population estimates come from the National Records of Scotland (NRS). Mortality rates are standardised rates per 100,000 population, using the 2013 European Standard Population [23]. A total of 59,241 deaths for men, aged 15–44 between 1980 and 2018 were included in our study (annual mean = 1519, sd = 120.4). Since the number of deaths per year is relatively small, 3-year rolling averages are used to describe trends in mortality.

For inequality analysis, deprivation was measured at the small-area level using population weighted deprivation quintiles of the Scottish Index of Multiple Deprivation (SIMD) for the years 2004, 2006, 2009, 2012, and 2016 [24]. The population weighted quintiles were calculated such that each quintile includes approximately 20% of the population. The 2004–2012 versions of the SIMD were provided for 2001 datazones (population mean = 815, sd = 275), and the 2016 version for the updated 2011 datazones (mean = 759, sd = 176). Deaths were combined into three-year groups (from 2001 to 2003 to 2016–2018) and linked to the closest SIMD version, with 2016 SIMD used for deaths from both 2013–2015 and 2016–2018.

Socioeconomic inequalities in health are measured using the slope index of inequality (SII) and the linear relative index of inequality (RII_L_). Both absolute and relative measures are important for public health monitoring [25]. Reductions in absolute inequalities imply overall health improvement and declines in mortality for all socioeconomic groups. However, this may still mean that the health of the better off is improving faster compared to the health of the worse off. A reduction in the relative inequalities will indicate that the health of the worse off population is improving relative faster compared to the population as a whole [26]. In addition, relative measures are more useful when comparing inequalities across time, age and cause of death, as is the case here. The confidence intervals (CIs) for both measures are calculated using a multinomial simulation method [27].

A number of different methods can be used to calculate both the slope and the relative indices [28]. Here, the SII is calculated using linear regression and the linear RII_L_ is the SII divided by the mean level of mortality across all socioeconomic groups [29]. The linear method chosen for the RII_L_ has the benefit of allowing the decomposition of inequalities by causes of death [28]. The decomposition of SII is more common, but as absolute inequalities increase with age, the decomposition of the RII_L_ is preferable when comparing inequalities across age groups.

The main cause of death was coded based on International Classification of Disease (ICD-9 for 1980–1999 and ICD-10 from 2000) with the exact codes show in supplement Table S1. Results are provided for different external causes of deaths and for leading internal causes of deaths. The categories were designed to be mutually exclusive, e.g. suicides (including undetermined intent) due to drugs are only included among suicides.

We have grouped the main causes of death into two broad categories of “external” and “internal” causes of mortality. The former includes ICD-10 chapter 20 “External causes of morbidity” together with alcohol and drug related deaths. Causes of death listed in the “internal” category are those with direct links to internal pathology rather than external actions. This dichotomy does not account for the impact external environment and behavioural factors have on many of the causes of death listed within the internal group (e.g. smoking and diet on cancers and heart disease). For this reason, the rates for external mortality as a whole are likely to be an underestimate. A small number of deaths from rare causes not presented in Table S1 are grouped under “other internal” (includes e.g. blood, endocrine and eye diseases).

### Role of the funding source

The sponsors of the study had no role in study design, data collection, analysis, interpretation, or writing of the report.

## Results

### Mortality rates by internal and external causes, 1980–2018

**Fig. 1:**
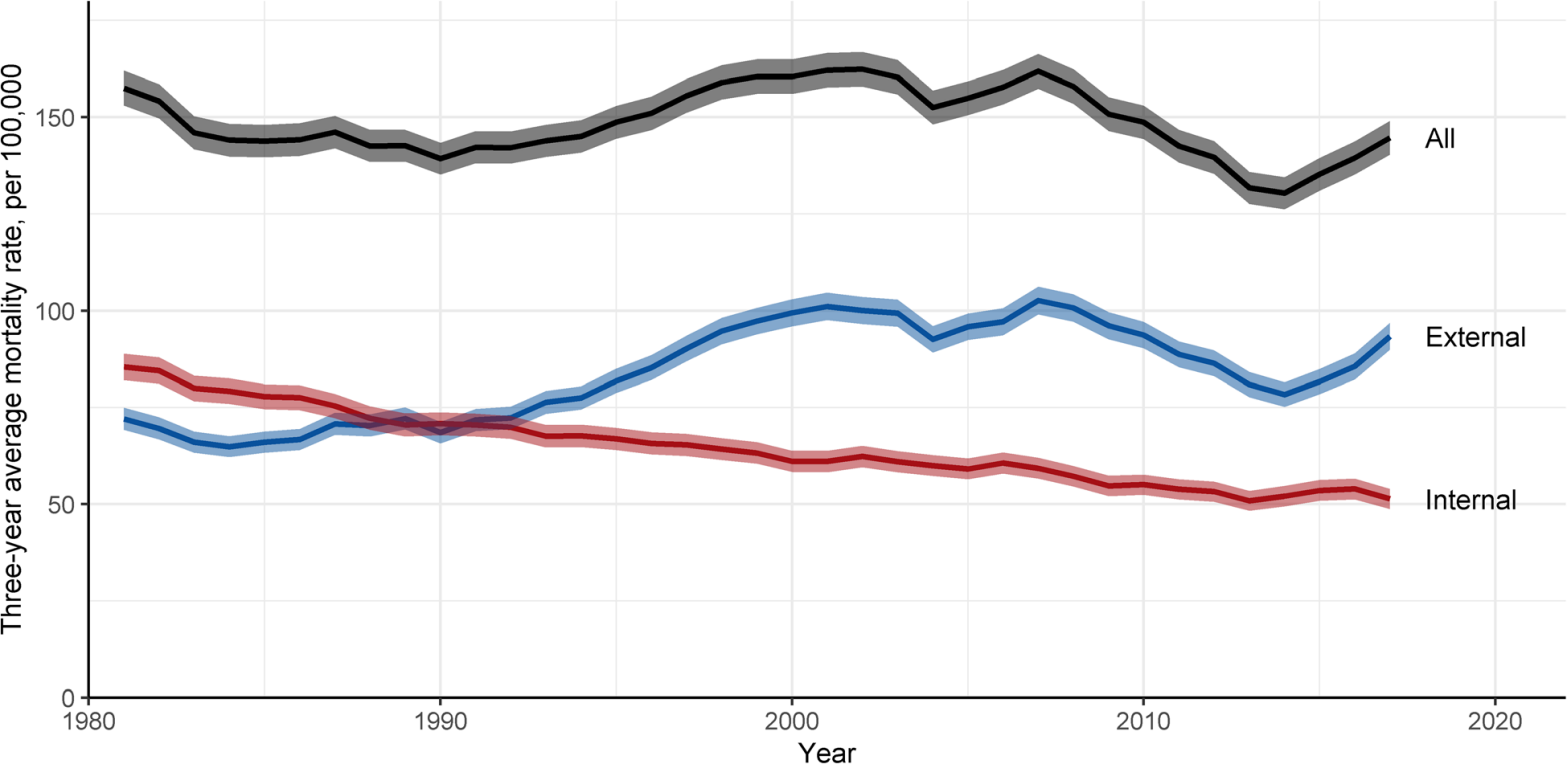
Mortality rate (with 95% CI) among men aged 15–44 by internal and external causes, 1980–2018

**Fig. 2:**
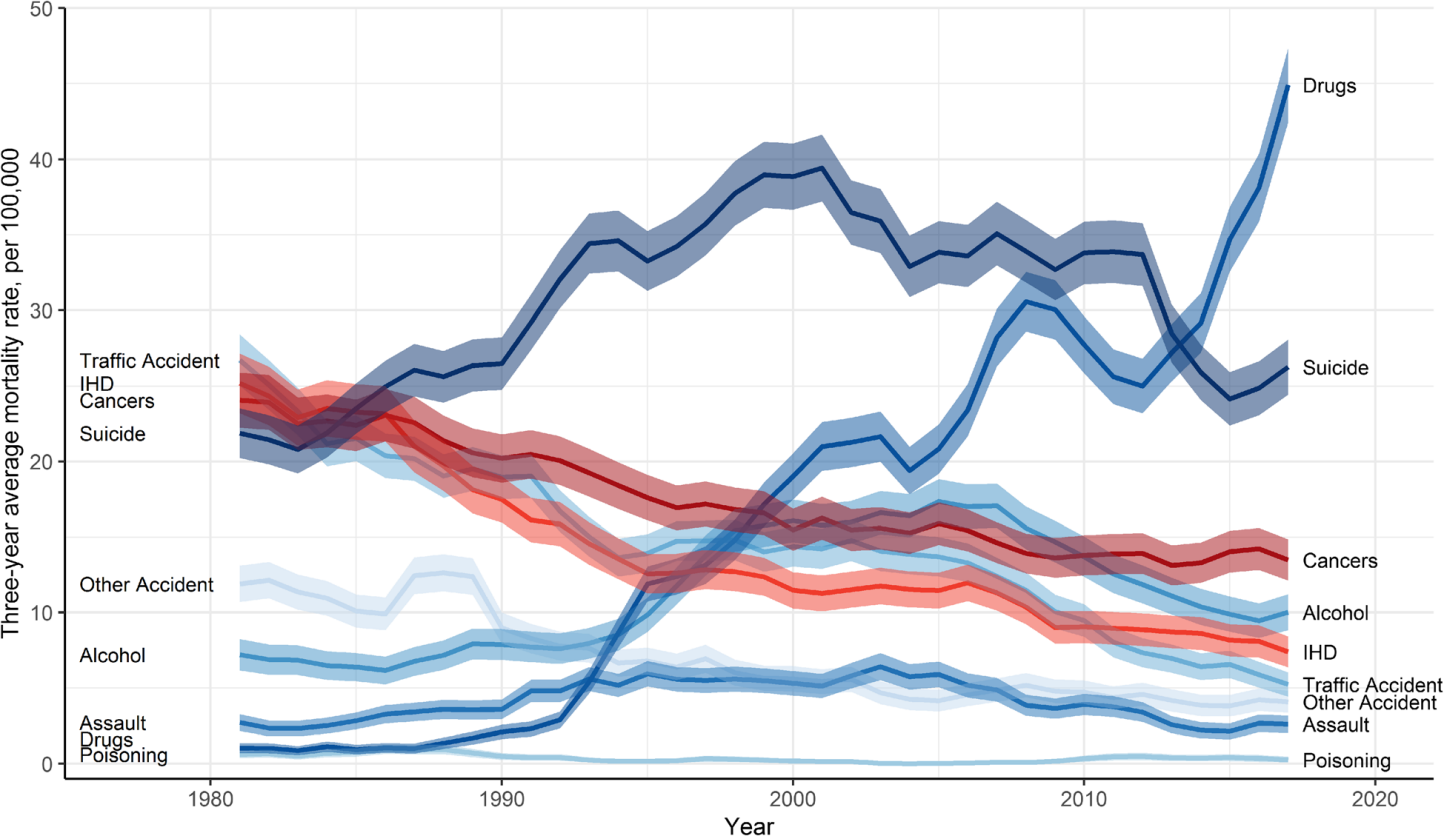
Mortality rate (with 95% CI) among men aged 15–44 by external causes and two leading internal causes, 1980–2018

Figure 1 shows an increase in mortality from external causes since the mid-1980s. While initially the increase was slow, it accelerated in the early 1990s and reached a peak in the 2000s. Over the past 20 years mortality from external causes has fluctuated, declining briefly around 2004 and then showing a more prolonged decline between 2007 and 2014. Since then, however, mortality from external causes has increased sharply. Contrary to these trends, mortality from internal causes has steadily declined, mainly due to reductions in mortality from IHD and cancers (Fig. 2). Trends in all-cause mortality shown in Fig. 1 follow that of external causes of death, i.e. the overall mortality for this age group of men is overwhelmingly shaped by external causes of death.

As shown in Fig. 2, the patterns in external causes of mortality are largely driven by drug-deaths and suicides. For the majority of the period, suicides have been the most dominant external cause, and also an overall leading cause of death. Suicides declined between 2001 and 2015, but in recent years have again increased. From 2013 drug-related deaths have become the leading external and overall cause of death for this age group of men. Drug-deaths have increased since the early 1990s almost yearly, but the recent rise since 2012 has been very sharp (approximately 80% between 2012 and 2018). Overall, drug-deaths account for around 50% of all external deaths in 2018 compared to just 3% in 1981.

Alcohol-related mortality increased from mid-1990s to 2007 but has since declined. It remains the third leading external and fourth overall cause of death. Deaths from assaults doubled between 1980 and 2005 but have since decreased and in 2018 were at the same level as three decades earlier. Mortality from traffic and other accidents has declined, and mortality from poisonings has remained relatively unchanged.

### Socioeconomic inequalities in mortality, 2001–2018

**Fig. 3:**
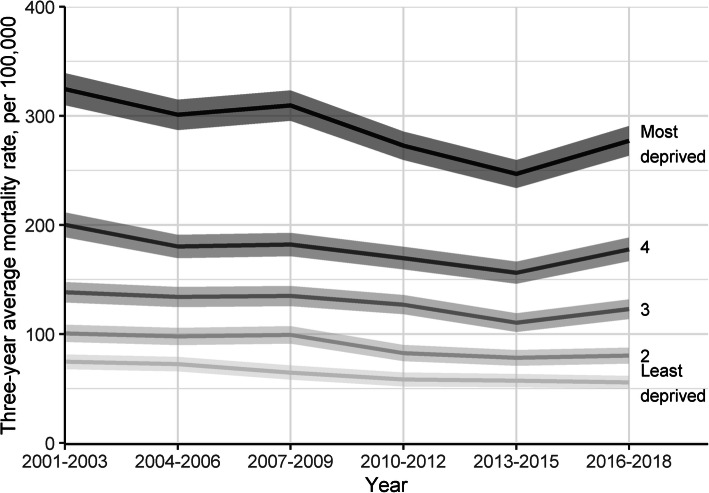
Mortality rate (with 95% CI) among men aged 15–44 by deprivation quintiles, 2001–2018

Figure 3 shows all-cause mortality from 2001 to 2018 by deprivation quintiles. (See Supplement Table S2 for mortality rates by deprivation for external, internal and five leading causes separately.) Between 2001 and 2015 mortality rates fell in all deprivation categories and absolute inequalities in mortality declined from SII = 304.9 (95% CI 288.5–319.8) in 2001–2003 to SII = 234.4 (221.1–248.7) in 2013–2015. This means that absolute difference in mortality rates between the most and least deprived areas declined by around 70 deaths per 100,000 in that period. However, between 2013 and 2015 and 2016–2018 mortality in quintiles 3 through 5 (most deprived) has increased, with bigger increases in the most deprived areas, leading to increased absolute inequalities (SII = 274.8 in 2016–2018; 95% CI 260.4–288.3).

The trends in mortality rates in Fig. 3 and the particularly the recent increase in absolute inequalities are driven by external causes of mortality. Inequalities from internal causes have continued to decline between 2013 and 2015 and 2016–2018. As a result, reductions in absolute inequalities between 2001 and 2003 to 2016–2018 have been relatively greater for internal causes of mortality and smaller for in external causes (Table 1). There has been a small increase in relative inequalities across all causes, driven by an increase in relative inequalities from external causes of mortality. Relative inequalities from internal causes of mortality have declined slightly.

**Table 1:** Absolute and relative inequalities in mortality by cause, 2001–2003 and 2016–2018

Cause	2001–2003					2016–2018				
	N	SII	95% CI	RII_L_	95% CI	N	SII	95% CI	RII_L_	95% CI
All	4965	301.5	(286.1–318)	1.84	(1.79–1.89)	4126	277.0	(263.1–290.7)	1.94	(1.9–1.97)
External	3058	209.4	(197.4–221.4)	1.28	(1.24–1.31)	2691	197.6	(186.3–208.9)	1.38	(1.34–1.42)
Internal	1907	92.1	(81.8–101.5)	0.56	(0.51–0.6)	1435	79.4	(70.4–87.6)	0.56	(0.51–0.59)
External causes										
Drugs	644	65.4	(60.6–70.1)	0.40	(0.38–0.42)	1281	120.9	(113.9–128.3)	0.85	(0.82–0.87)
Suicide	1112	62.9	(55.7–69.7)	0.38	(0.35–0.41)	774	34.1	(28.1–39.9)	0.24	(0.2–0.27)
Alcohol	491	50.1	(45.9–54.2)	0.31	(0.29–0.32)	270	25.0	(21.3–28.4)	0.17	(0.15–0.19)
Assault	179	20.9	(18.6–23.1)	0.13	(0.12–0.14)	75	7.9	(6.4–9.2)	0.06	(0.05–0.06)
Other accidents^a^	175	8.7	(5.9–11.4)	0.05	(0.04–0.07)	127	6.1	(3.0–9.0)	0.04	(0.02–0.05)
Traffic accidents	457	1.4	(−3.2–6.6)	0.01	(−0.02–0.04)	164	3.7	(1–6.5)	0.03	(0.01–0.04)
Internal causes										
IHD	348	20.6	(16.8–24.3)	0.13	(0.11–0.14)	202	16.1	(13.2–19)	0.11	(0.1–0.13)
Cancers	477	8.8	(4–13.7)	0.05	(0.03–0.08)	381	10.6	(5.5–15.4)	0.07	(0.04–0.1)
Respiratory disease	100	8.3	(6.2–10.1)	0.05	(0.04–0.06)	82	5.2	(3.4–6.9)	0.04	(0.02–0.05)
Diabetes	53	2.9	(1.7–4.1)	0.02	(0.01–0.02)	73	5.2	(3.4–7.1)	0.04	(0.02–0.05)
Digestive system disease	115	8.0	(5.8–10.1)	0.05	(0.04–0.06)	66	4.7	(2.8–6.3)	0.03	(0.02–0.04)
Nervous system disease	98	3.9	(1.8–6.1)	0.02	(0.01–0.04)	100	3.5	(1.4–5.7)	0.02	(0.01–0.04)
Epilepsy	97	6.1	(4–8.3)	0.04	(0.03–0.05)	49	3.4	(1.7–4.9)	0.02	(0.01–0.03)
Infections	114	7.7	(5.6–9.9)	0.05	(0.03–0.06)	38	2.9	(1.6–4.2)	0.02	(0.01–0.03)
CBVD	106	6.1	(3.7–8.3)	0.04	(0.02–0.05)	53	2.7	(0.9–4.5)	0.02	(0.01–0.03)
Other	399	19.7	(15.6–23.7)	0.12	(0.1–0.14)	391	25.1	(21–29.3)	0.18	(0.15–0.2)

When RII_L_ is 0, there are no inequalities in mortality and values above zero indicate higher mortality in deprived areas relative to the overall mortality across all socioeconomic groups. An RII_L_ value of 1 suggests that mortality rates in the most deprived areas are about 50% above average. The maximum value of RII_L_ is approximately 2, but it may exceed this if inequalities are very high

^a^ Includes accidental poisoning, 5 cases in 2001–2003 and 8 in 2016–2018

As shown in Table 1, the trends in inequalities are different for the specific causes. Both absolute and relative inequalities for drug-related mortality have doubled over the period, while inequalities for most other external causes have reduced. Notably, between 2001 and 2003 to 2010–2012 suicides contributed to inequalities equally with drug-deaths, but in 2016–2018 the contribution of suicides to inequalities (SII = 34.1; 95% CI 28.1–39.9) is almost a quarter of that of drug-deaths (SII = 120.9; 113.9–128.3). Reductions in both absolute and relative inequalities have also been evident for alcohol mortality and assaults, but inequalities are now apparent for traffic accidents (Table 1).

Together, deaths from drugs, alcohol and suicides made up 65% of absolute inequalities in mortality in 2016–2018. In 2001–2003 the same three causes contributed 59% of absolute inequalities in mortality. While the relative contributions of the three causes to absolute inequalities have changed, their combined effect has remained consistently around 60% between 2001 and 2003 and 2016–2018 (Supplement Table S2).

Absolute inequalities for IHD and respiratory disease have reduced slightly, but inequalities in cancers and diabetes have increased. Relative inequalities for any of the internal causes of mortality have not changed notably. Overall, internal causes of death contribute much less to inequalities in mortality among men aged 15–44 compared to external causes of death.

**Fig. 4:**
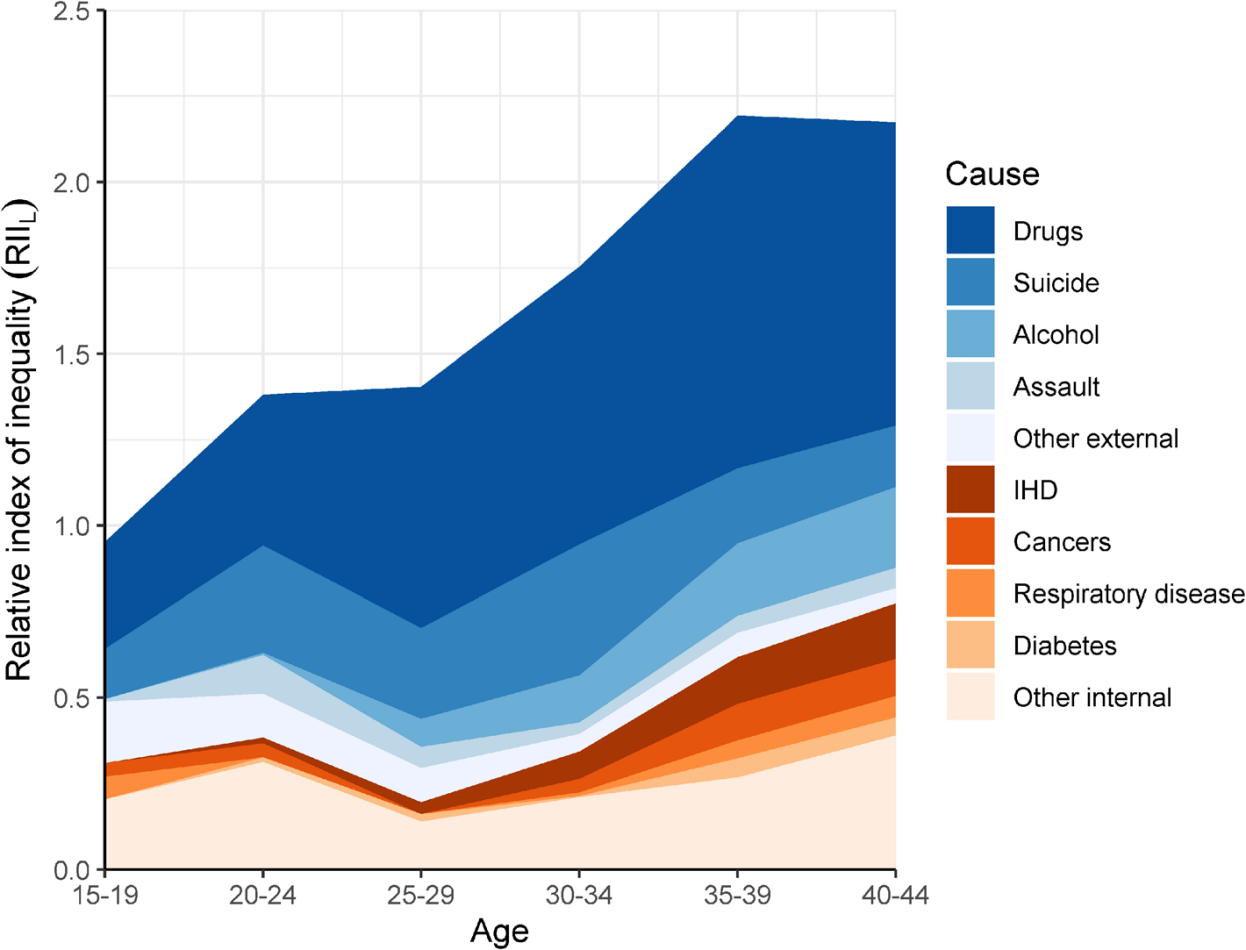
Relative inequalities (RII_L_) in mortality by cause and age group, 2016–2018

Figure 4 shows the decomposed RII_L_ for the main external and internal causes of death by 5-year age groups in 2016–2018. The height of the plot indicates the overall RII_L_ for all causes of deaths. Relative inequalities in mortality increase with age and are highest for ages 40–44 in this study of 15–44 year olds. There is some variation in the role of the different causes of death to relative inequalities across 5-year age groups. Depending on the age group, drug deaths contribute 25–45% of the socioeconomic inequalities in young men. At ages 20–24 assaults contribute more to relative inequalities than alcohol, IHD or cancers, and the leading internal causes of mortality generally become more significant after the age of 30. Suicides, however, have a fairly consistent contribution to relative inequalities across different ages.

## Discussion

For nearly four decades the two leading causes of death and also the leading contributors to inequalities in mortality among men aged 15–44 in Scotland have both been external causes - drug-related deaths and suicides. Suicide death nearly doubled between 1980 and 2000 and were the dominant cause of death from 1985 to 2012 among this population group. Drug deaths increased rapidly since 1992 and in 2013 overtook suicides as the single leading cause in mortality. Today, mortality from drugs is the biggest contributor to socioeconomic inequalities in mortality among young men in Scotland (Fig. 2 and Table 1). The periodic fluctuations in drug and suicide mortality may be explained by cohort effects, such as among men most affected by the neoliberal economic policies of the 1980s [30, 31], and the temporary decline in drug mortality between 2009 and 2012 could be related to the “heroin drought” of 2010/2011 [32]. However, our aim here is to highlight that mortality among men aged 15–44 from external causes and from deaths of despair overall has increased since 1980 and is not displaying a sustained decline (Fig. 1).

Increasing mortality among young and middle-aged men, and the role of drug deaths have been noted internationally [3, 4, 6], but drug mortality in Scotland still stands out as exceptionally high in comparison to England and the rest of Europe [20]. Importantly, rising mortality rates among young men in Scotland and the contribution of suicides, drugs and alcohol to this was already noted in the early 2000’s [18], more than a decade before the deaths of despair received heightened media attention in the US.

Despite receiving less international attention, the trends and prevalence of drug deaths in Scotland are similar to those in the US. By the late 1990s there were clear signs of an increasing drug problem in both countries [7, 18] and drug mortality has continued to rise since, with a more rapid increase from 2013. Deaths are especially high among men and ages 25–54 [20, 33]. In the US, the rates and increases are also high among ages 55–64 [33], while in Scotland the largest increases in recent years have been among ages 35–54 [20]. In both countries drug deaths now surpass suicides and alcohol mortality among young and middle-aged men [7] and are more likely to affect those of lower SES [6].

Different hypotheses, ranging from genetics, migration, health behaviours to economic policies and a “political attack”, have been proposed to explain high mortality rates in Scotland [34]. By examining the different causes of death among a specific demographic group and by deprivation levels, we can say which explanations are more likely than others. The causes driving mortality and inequalities in mortality among young men are not directly related to genetic differences, migration, poor diet, or lack of exercise. High rates of mortality from alcohol, suicides, and drugs, especially in the more deprived areas, can be better explained by theories emphasizing marginalization - lack of power (political and economic), opportunities and social support structures to overcome adverse life events. They speak of despair, of self-destructive behaviours that can have a direct effect on mortality through suicide, or an indirect impact through substance abuse [6].

Since the 1980s the Scottish economy has undergone major changes, such as the substantial loss of heavy industry jobs, and the financial crisis of 2007 and subsequent imposition of austerity measures, significantly affecting employment and income levels. The links between economic opportunities and mortality from drugs, alcohol and suicide have been made before. The impact of economic recessions on suicides and alcohol abuse is well documented [35, 36] and evidence also suggest increased disorders from illicit drug use during economic downturns [37]. In addition, those of lower SES may be more likely to experience depression or substance use disorders during recessions [37, 38], supporting our findings of high socio-economic inequalities in mortality from suicides, drugs and alcohol. The impact of economic change on health can also be very long-lasting. For example, the cohort of men born in Scotland between 1960 and 1980 and affected by the neoliberal policies of the 1980s have experienced high drug and suicide mortality also in the 1990s and early 2000s, decades after the economic changes [30, 31].

Men may also be more adversely affected by the transition to the service economy compared to women. Young men are excluded and exclude themselves from the service sector labour due to prominent societal stereotypes of masculinity and the types of jobs regarded as appropriate for men [39, 40]. Qualitative research has shown that for men, service jobs can lead to social isolation such as rejection by peers and the family, despair and to depressive symptoms [39]. Again, men of lower SES are most affected by this economic transition and the associated stereotypes as they appear to lack the “deferential servility” required in the service economy [40]. To summarise, our evidence on high mortality and inequalities in mortality from drugs, suicides and alcohol among men aged 15–44 closely ties in with the body of evidence showing that economic recession and transition to the service economy adversely affect mental health, and that young men of low SES are particularly affected.

Due to the potential years of life and economic productivity lost, the economic burden of deaths among young men is high [21]. Together with the increasing old age dependency ratio (number of people over 65 for every 1000 people aged 16–64) and ageing population in Scotland and in the UK [41], rising mortality among young men is not only a major public health concern, but a serious looming social and economic problem. For these reasons, the trends in mortality and inequalities in mortality we present require immediate and decisive action that addresses the upstream determinants of health, something that has increasingly been called for also in England [14, 42].

Scotland has introduced separate policies to prevent suicides [43], tackle the drug-problem [44] and change its relationship with alcohol [45]. These policies mention the contribution of deprivation to self-harm and substance mis-use to a varying degree and, until very recently [46], have not been joined up or focused on reducing deprivation and socio-economic inequalities as common causes. As a result, suicide prevention policies may have been effective in reducing deaths from self-harm, but as the underlying causes, e.g. deprivation, have not been sufficiently addressed, mortality has increased from other external causes, such as drugs. Unlike in the USA, drug mortality in Europe (and Scotland) is not driven by the illicit use of prescription pain medication [47]. Heroin and methadone still contribute to the vast majority of drug deaths in Scotland [20], providing little evidence that drug mortality has increased due to the rising availability, over-prescription and misuse of opioid-based painkillers among medical practitioners. As such, policies to reduce drug deaths that focus solely on reducing access to drugs might not be very effective. Instead, a joined-up framework focusing on the common causes (e.g. social determinants of health) leading to the deaths of despair can be more effective in reducing mortality and inequalities in mortality as a whole.

Deaths of despair are not an inevitability of economic transitions or recessions. Countries with high investment in social protection and active labour market programs do not experience increases in suicides or alcohol abuse during recessions [36].

## Strengths and limitations

Deaths at ages 15–44 are relatively uncommon and this can sometimes limit the analysis. We have overcome the small numbers issue by using population level data over nearly four decades and grouping years (e.g. using rolling averages) to provide more robust evidence. This has enabled us to examine of a wide range of causes of death relevant to younger age groups. Deprivation is measured at the small-area level as an individual level measure is not available. While the death records include the National Statistics Socio-economic Classification (NS-SEC), only 72% of men aged 15–44 have a valid value recorded, and education data can be linked with mortality only for the youngest in our cohort. For some men the individual level measure of SES will not match area deprivation. However, Scottish datazones used in this study are half the size of the Scottish postcode sectors and the English lower-layer super output areas, increasing the probability that datazones are homogenous. In addition, we expect an individual level measure of SES to produce higher rather than lower inequalities, meaning that our results are more likely to be an under- rather than an overestimate.

In the US, the argument that deaths from drugs, alcohol and suicide are caused by despair has been contested by longer time series analysis and the suggestion that the recent increases in mortality among young and middle-aged men are more strongly associated with the opioid epidemic [7]. In this study we observe trends in mortality over four decades and show that all three causes (alcohol, suicide and drugs) have contributed substantially to mortality and to inequalities in mortality among men. As discussed above, there is also no evidence of a concurrent opioid epidemic in Scotland (unlike in the US) that could independently explain the increase in drug mortality.

## Conclusions

By exploring long-term patterns in cause-specific mortality and inequalities in mortality, our study highlights the immediate need for policies and interventions to reduce mortality and health inequalities among young men in Scotland. While significant progress has been made over the last 40 years towards reducing mortality from internal causes such as heart disease and cancers, this has not resulted in similar reductions in overall mortality because of the increases in deaths from suicides, alcohol-harm and in recent years particularly from drugs. Deaths from these causes can and should be prevented. However, preventing deaths that are so tightly related to socioeconomic status require policy interventions that not only tackle the mental health or addiction problems, but also intergenerational deprivation, socioeconomic inequalities, and a lack of opportunities for upward social mobility.

## Supplementary Information

**Additional file 1: Table S1.** Classification of main causes of death. **Table S2.** Mortality rates by deprivation quintiles and inequalities in mortality from 2001 to 2003 to 2016–2018 for five main causes of death among men aged 15–44.

## Data Availability

The data that support the findings of this study are available from National Records of Scotland (NRS) but restrictions apply to the availability of these data, which were used under license for the current study, and so are not publicly available. Data are however available from the authors upon reasonable request and with permission of NRS.

## Abbreviations

SII: Slope index of inequality
RII_L_: Relative index of inequality (linear)
