# Supplementary material for: Deaths of despair: cause-specific mortality and socioeconomic inequalities in cause-specific mortality among young men in Scotland
Source: Int J Equity Health. 2020 Dec 4;19:215. doi: 10.1186/s12939-020-01329-7 (PMC7716282; doi:10.1186/s12939-020-01329-7)
Supplement: Supplementary file 1 — Additional file 1: Table S1. Classification of main causes of death. Table S2. Mortality rates by deprivation quintiles and inequalities in mortality from 2001 to 2003 to 2016–2018 for five main causes of death among men aged 15–44. [file 12939_2020_1329_MOESM1_ESM.docx]

**Supplement**

**Table S.1 Classification of main causes of death**

| **External causes** | **ICD-9 codes** | **ICD-10 codes** |  | **Internal causes** | **ICD-9 codes** | **ICD-10 codes** |
| --- | --- | --- | --- | --- | --- | --- |
| Drug-related | 292, 304, 305.2-305.9, E850-E858 | F11-F16, F18-F19, X40-X44 |  | Ischaemic heart disease (IHD) | 410-414 | I20-I25 |
| Suicide | E950-E959, E980-E989 | X60-X84, Y10-Y34, Y87.0, Y87.2 |  | Malignant neoplasms | 140-208 | C00-C97 |
| Alcohol-related | 291, 303, 305.0, 357.5, 425.5, 535.3, 571 (excluding 571.6), E860 | F10, K70, K73, X45, G31.2, G62.1, I42.6, K29.2, K74.0-K74.2, K74.6, K86.0 |  | Respiratory disease | 460-517 | J00-J99 |
| Assault | E960-E969 | X85-Y09 |  | Diabetes mellitus | 250 | E10-E14 |
| Traffic accidents | E800-E848 | V01-V99 |  | Digestive system disease (excluding alcohol) | 520-579 (excluding alcohol-related) | K00-K93 (excluding alcohol-related) |
| Accidental poisoning | E861-E869 | X46-X49 |  | Epilepsy | 345 | G40 |
| Other accidents | E849-E928 (excluding E850-E879) | W00-X30, X50 |  | Nervous system disease (excluding epilepsy) | 320-359 (excluding 345 and 357.5) | G00-G99 (excluding G40 and G31.2, G62.1) |
|  |  |  |  | Infectious diseases | 001-139 | A00-B99 |
|  |  |  |  | Stroke | 430-438 | I60-I69 |

**Table S.2 Mortality rates by deprivation quintiles and inequalities in mortality from 2001-2003 to 2016-2018 for five main causes of death among men aged 15-44**

|  | 2001-2003 | |  | 2004-2006 | |  | 2007-2009 | |  | 2010-2012 | |  | 2013-2015 | |  | 2016-2018 | |
| --- | --- | --- | --- | --- | --- | --- | --- | --- | --- | --- | --- | --- | --- | --- | --- | --- | --- |
| Deprivation | Value | 95% CI |  | Value | 95% CI |  | Value | 95% CI |  | Value | 95% CI |  | Value | 95% CI |  | Value | 95% CI |
| External |  |  |  |  |  |  |  |  |  |  |  |  |  |  |  |  |  |
| Least deprived | 42.5 | (37.4-47.7) |  | 39.7 | (34.6-44.7) |  | 37.4 | (32.4-42.5) |  | 33.6 | (28.7-38.4) |  | 29.2 | (24.8-33.5) |  | 29.4 | (25-33.7) |
| 2 | 55.9 | (49.9-61.8) |  | 56.5 | (50.4-62.6) |  | 62.5 | (56.1-68.9) |  | 48.4 | (42.7-54.1) |  | 45.8 | (40.3-51.4) |  | 49.3 | (43.6-55) |
| 3 | 81.9 | (74.6-89.1) |  | 81.6 | (74.4-88.8) |  | 81.6 | (74.4-88.7) |  | 74.0 | (67.3-80.8) |  | 65.6 | (59.1-72.2) |  | 76.3 | (69.2-83.4) |
| 4 | 118.0 | (109.3-126.7) |  | 111.8 | (103.4-120.2) |  | 114.6 | (106.1-123) |  | 103.6 | (95.5-111.7) |  | 92.5 | (84.8-100.3) |  | 114.9 | (106.2-123.6) |
| Most deprived | 220.0 | (207.8-232.1) |  | 198.5 | (187.1-209.8) |  | 207.2 | (195.7-218.6) |  | 181.6 | (170.9-192.3) |  | 154.6 | (144.5-164.6) |  | 188.9 | (177.9-200) |
| Overall | 101.6 | (98-105.2) |  | 97.2 | (93.7-100.7) |  | 101.3 | (97.7-104.9) |  | 89.5 | (86.1-92.9) |  | 78.0 | (74.8-81.2) |  | 92.3 | (88.8-95.8) |
|  |  |  |  |  |  |  |  |  |  |  |  |  |  |  |  |  |  |
| SII | 209.4 | (197.4-221.4) |  | 189.4 | (178.8-201.4) |  | 198.8 | (187.9-210.7) |  | 178.6 | (167.1-190.1) |  | 153.9 | (143.4-163.8) |  | 197.6 | (186.3-208.9) |
| RII_L_ | 1.28 | (1.24-1.31) |  | 1.21 | (1.18-1.25) |  | 1.26 | (1.22-1.29) |  | 1.24 | (1.2-1.29) |  | 1.18 | (1.14-1.22) |  | 1.38 | (1.34-1.42) |
| % from total SII | 69.5 |  |  | 69.2 |  |  | 68.7 |  |  | 68.2 |  |  | 65.2 |  |  | 71.3 |  |
|  |  |  |  |  |  |  |  |  |  |  |  |  |  |  |  |  |  |
| Internal |  |  |  |  |  |  |  |  |  |  |  |  |  |  |  |  |  |
| Least deprived | 32.1 | (27.7-36.4) |  | 32.7 | (28.3-37.2) |  | 27.3 | (23.1-31.5) |  | 24.8 | (20.6-28.9) |  | 28.1 | (23.8-32.4) |  | 26.2 | (22.1-30.4) |
| 2 | 44.8 | (39.5-50) |  | 41.4 | (36.3-46.4) |  | 36.8 | (32-41.6) |  | 34.2 | (29.4-38.9) |  | 32.4 | (27.7-37) |  | 31.0 | (26.4-35.5) |
| 3 | 56.4 | (50.4-62.4) |  | 52.4 | (46.7-58.2) |  | 53.3 | (47.6-59.1) |  | 53.0 | (47.2-58.7) |  | 44.8 | (39.3-50.3) |  | 46.5 | (40.9-52.2) |
| 4 | 82.2 | (74.9-89.4) |  | 68.6 | (62-75.2) |  | 67.5 | (61-74) |  | 66.0 | (59.5-72.5) |  | 63.7 | (57.1-70.2) |  | 62.9 | (56.3-69.4) |
| Most deprived | 104.5 | (96.1-113) |  | 102.6 | (94.4-110.8) |  | 102.3 | (94.2-110.4) |  | 91.0 | (83.4-98.7) |  | 92.2 | (84.3-100.1) |  | 88.3 | (80.5-96.1) |
| Overall | 62.4 | (59.6-65.2) |  | 58.9 | (56.1-61.6) |  | 57.1 | (54.4-59.8) |  | 54.1 | (51.4-56.7) |  | 51.9 | (49.3-54.6) |  | 50.8 | (48.1-53.4) |
|  |  |  |  |  |  |  |  |  |  |  |  |  |  |  |  |  |  |
| SII | 92.1 | (81.8-101.5) |  | 84.4 | (74.5-93.3) |  | 90.5 | (81.7-98.8) |  | 83.4 | (74.1-91.8) |  | 82.1 | (73.5-91.3) |  | 79.4 | (70.4-87.6) |
| RII_L_ | 0.56 | (0.51-0.6) |  | 0.54 | (0.49-0.58) |  | 0.57 | (0.53-0.61) |  | 0.58 | (0.53-0.62) |  | 0.63 | (0.58-0.68) |  | 0.56 | (0.51-0.59) |
| % from total SII | 30.5 |  |  | 30.8 |  |  | 31.3 |  |  | 31.8 |  |  | 34.8 |  |  | 28.7 |  |
|  |  |  |  |  |  |  |  |  |  |  |  |  |  |  |  |  |  |
| Drugs |  |  |  |  |  |  |  |  |  |  |  |  |  |  |  |  |  |
| Least deprived | 4.9 | (3.1-6.7) |  | 4.1 | (2.5-5.8) |  | 7.5 | (5.2-9.8) |  | 5.7 | (3.7-7.8) |  | 6.1 | (4.1-8.2) |  | 6.8 | (4.7-8.9) |
| 2 | 7.8 | (5.6-10.1) |  | 7.7 | (5.4-10) |  | 11.5 | (8.7-14.2) |  | 10.4 | (7.7-13) |  | 13.4 | (10.4-16.4) |  | 18.0 | (14.5-21.5) |
| 3 | 10.3 | (7.7-12.9) |  | 14.6 | (11.5-17.6) |  | 23.6 | (19.8-27.5) |  | 18.3 | (14.9-21.7) |  | 22.4 | (18.6-26.3) |  | 34.3 | (29.5-39.1) |
| 4 | 24.6 | (20.6-28.5) |  | 25.9 | (21.9-30) |  | 36.2 | (31.5-41) |  | 30.0 | (25.6-34.3) |  | 31.7 | (27.2-36.3) |  | 56.0 | (49.9-62.1) |
| Most deprived | 61.2 | (54.8-67.5) |  | 51.1 | (45.4-56.8) |  | 70.5 | (63.9-77.2) |  | 61.0 | (54.8-67.2) |  | 70.1 | (63.3-76.8) |  | 106.2 | (97.8-114.6) |
| Overall | 21.5 | (19.8-23.2) |  | 20.9 | (19.2-22.5) |  | 30.4 | (28.4-32.3) |  | 25.6 | (23.8-27.4) |  | 29.0 | (27.1-31) |  | 44.5 | (42.1-47) |
|  |  |  |  |  |  |  |  |  |  |  |  |  |  |  |  |  |  |
| SII | 65.4 | (60.6-70.1) |  | 57.8 | (52.9-62.2) |  | 77.9 | (72-83.7) |  | 66.8 | (61.6-72) |  | 76.3 | (70.2-81.8) |  | 120.9 | (113.9-128.3) |
| RII_L_ | 0.40 | (0.38-0.42) |  | 0.37 | (0.35-0.39) |  | 0.49 | (0.47-0.51) |  | 0.47 | (0.44-0.49) |  | 0.59 | (0.56-0.61) |  | 0.85 | (0.82-0.87) |
| % from total SII | 21.7 |  |  | 21.1 |  |  | 26.9 |  |  | 25.5 |  |  | 32.3 |  |  | 43.6 |  |
|  |  |  |  |  |  |  |  |  |  |  |  |  |  |  |  |  |  |
| Suicides |  |  |  |  |  |  |  |  |  |  |  |  |  |  |  |  |  |
| Least deprived | 17.0 | (13.7-20.3) |  | 15.3 | (12.2-18.5) |  | 14.7 | (11.6-17.9) |  | 15.9 | (12.6-19.3) |  | 14.3 | (11.2-17.3) |  | 13.7 | (10.7-16.6) |
| 2 | 22.4 | (18.6-26.1) |  | 25.2 | (21.2-29.2) |  | 24.6 | (20.6-28.6) |  | 17.4 | (14-20.8) |  | 17.6 | (14.1-21) |  | 18.9 | (15.4-22.4) |
| 3 | 35.2 | (30.4-39.9) |  | 30.5 | (26.1-34.9) |  | 26.6 | (22.6-30.7) |  | 32.9 | (28.4-37.4) |  | 23.6 | (19.7-27.5) |  | 23.3 | (19.4-27.2) |
| 4 | 42.6 | (37.4-47.9) |  | 41.2 | (36.1-46.3) |  | 38.9 | (34-43.9) |  | 39.5 | (34.5-44.5) |  | 32.7 | (28.1-37.3) |  | 32.8 | (28.2-37.4) |
| Most deprived | 69.8 | (63-76.6) |  | 59.0 | (52.8-65.2) |  | 64.0 | (57.6-70.3) |  | 63.0 | (56.8-69.3) |  | 40.4 | (35.4-45.5) |  | 39.1 | (34.2-44) |
| Overall | 36.9 | (34.8-39.1) |  | 34.3 | (32.2-36.4) |  | 34.1 | (32-36.2) |  | 34.3 | (32.2-36.4) |  | 25.9 | (24-27.7) |  | 25.8 | (23.9-27.6) |
|  |  |  |  |  |  |  |  |  |  |  |  |  |  |  |  |  |  |
| SII | 62.9 | (55.7-69.7) |  | 52.3 | (44.8-58.5) |  | 55.8 | (49-62.5) |  | 58.2 | (50.9-64.9) |  | 34.7 | (28.5-40.3) |  | 34.1 | (28.1-39.9) |
| RII_L_ | 0.38 | (0.35-0.41) |  | 0.33 | (0.3-0.36) |  | 0.35 | (0.32-0.38) |  | 0.40 | (0.37-0.44) |  | 0.27 | (0.23-0.3) |  | 0.24 | (0.2-0.27) |
| % from total SII | 20.9 |  |  | 19.1 |  |  | 19.3 |  |  | 22.2 |  |  | 14.7 |  |  | 12.3 |  |
|  |  |  |  |  |  |  |  |  |  |  |  |  |  |  |  |  |  |
| Alcohol |  |  |  |  |  |  |  |  |  |  |  |  |  |  |  |  |  |
| Least deprived | 3.4 | (2-4.8) |  | 3.2 | (1.8-4.6) |  | 1.9 | (0.8-3) |  | 1.7 | (0.6-2.8) |  | 1.4 | (0.4-2.3) |  | 2.5 | (1.2-3.8) |
| 2 | 4.6 | (2.9-6.2) |  | 4.9 | (3.2-6.6) |  | 5.8 | (3.9-7.7) |  | 3.8 | (2.3-5.4) |  | 5.7 | (3.7-7.7) |  | 3.9 | (2.3-5.6) |
| 3 | 9.0 | (6.7-11.4) |  | 11.6 | (8.9-14.3) |  | 11.0 | (8.4-13.6) |  | 7.9 | (5.7-10.2) |  | 6.4 | (4.3-8.5) |  | 8.1 | (5.7-10.5) |
| 4 | 22.1 | (18.3-25.9) |  | 20.1 | (16.5-23.6) |  | 18.2 | (14.8-21.5) |  | 16.1 | (12.9-19.4) |  | 15.0 | (11.8-18.3) |  | 11.8 | (8.9-14.7) |
| Most deprived | 45.2 | (39.6-50.8) |  | 49.9 | (44.1-55.6) |  | 41.7 | (36.5-47) |  | 31.8 | (27.3-36.4) |  | 23.5 | (19.5-27.6) |  | 23.1 | (19.1-27.1) |
| Overall | 15.9 | (14.5-17.3) |  | 17.3 | (15.9-18.8) |  | 15.5 | (14.1-16.9) |  | 12.4 | (11.1-13.7) |  | 10.4 | (9.2-11.6) |  | 9.9 | (8.7-11.1) |
|  |  |  |  |  |  |  |  |  |  |  |  |  |  |  |  |  |  |
| SII | 50.1 | (45.9-54.2) |  | 55.3 | (51-59.6) |  | 46.9 | (43.1-50.9) |  | 37.1 | (33.3-40.6) |  | 27.7 | (24-31.2) |  | 25.0 | (21.3-28.4) |
| RII_L_ | 0.31 | (0.29-0.32) |  | 0.35 | (0.34-0.37) |  | 0.30 | (0.28-0.31) |  | 0.26 | (0.24-0.27) |  | 0.21 | (0.19-0.23) |  | 0.17 | (0.15-0.19) |
| % from total SII | 16.6 |  |  | 20.2 |  |  | 16.2 |  |  | 14.1 |  |  | 11.7 |  |  | 9.0 |  |
|  |  |  |  |  |  |  |  |  |  |  |  |  |  |  |  |  |  |
| IHD |  |  |  |  |  |  |  |  |  |  |  |  |  |  |  |  |  |
| Least deprived | 4.0 | (2.5-5.5) |  | 3.7 | (2.2-5.1) |  | 3.7 | (2.2-5.2) |  | 3.0 | (1.6-4.5) |  | 3.1 | (1.7-4.6) |  | 1.3 | (0.3-2.2) |
| 2 | 7.6 | (5.5-9.7) |  | 7.3 | (5.3-9.4) |  | 5.3 | (3.6-7.1) |  | 3.5 | (2-5) |  | 4.5 | (2.7-6.2) |  | 4.7 | (2.9-6.5) |
| 3 | 9.6 | (7.1-12) |  | 9.1 | (6.7-11.4) |  | 10.9 | (8.3-13.5) |  | 9.5 | (7.1-12) |  | 7.3 | (5.1-9.5) |  | 6.1 | (4-8.2) |
| 4 | 17.4 | (14-20.7) |  | 15.1 | (12.1-18.2) |  | 11.7 | (8.9-14.4) |  | 12.0 | (9.2-14.8) |  | 9.7 | (7.1-12.3) |  | 10.8 | (8-13.6) |
| Most deprived | 19.5 | (15.8-23.2) |  | 22.2 | (18.4-26.1) |  | 20.5 | (16.8-24.1) |  | 16.6 | (13.3-19.9) |  | 18.1 | (14.6-21.6) |  | 14.2 | (11.1-17.4) |
| Overall | 11.2 | (10-12.4) |  | 11.2 | (10-12.4) |  | 10.3 | (9.2-11.4) |  | 9.0 | (7.9-10) |  | 8.5 | (7.4-9.5) |  | 7.4 | (6.4-8.4) |
|  |  |  |  |  |  |  |  |  |  |  |  |  |  |  |  |  |  |
| SII | 20.6 | (16.8-24.3) |  | 22.9 | (19.2-26.6) |  | 20.4 | (16.7-24) |  | 17.8 | (14.4-21.4) |  | 18.6 | (14.9-21.9) |  | 16.1 | (13.2-19) |
| RII_L_ | 0.13 | (0.11-0.14) |  | 0.15 | (0.13-0.17) |  | 0.13 | (0.11-0.15) |  | 0.12 | (0.10-0.14) |  | 0.14 | (0.12-0.16) |  | 0.11 | (0.10-0.13) |
| % from total SII | 6.8 |  |  | 8.3 |  |  | 7.1 |  |  | 6.8 |  |  | 7.9 |  |  | 5.8 |  |
|  |  |  |  |  |  |  |  |  |  |  |  |  |  |  |  |  |  |
| Cancers |  |  |  |  |  |  |  |  |  |  |  |  |  |  |  |  |  |
| Least deprived | 12.9 | (10.1-15.6) |  | 14.2 | (11.3-17.2) |  | 8.4 | (6.1-10.7) |  | 9.1 | (6.6-11.7) |  | 10.8 | (8.1-13.5) |  | 12.1 | (9.2-15) |
| 2 | 13.6 | (10.7-16.5) |  | 13.8 | (10.9-16.7) |  | 10.7 | (8.1-13.3) |  | 11.3 | (8.6-14.1) |  | 10.2 | (7.6-12.8) |  | 10.4 | (7.7-13) |
| 3 | 15.1 | (12-18.2) |  | 15.6 | (12.5-18.8) |  | 14.3 | (11.3-17.2) |  | 14.5 | (11.5-17.5) |  | 13.1 | (10.2-16.1) |  | 11.1 | (8.3-13.8) |
| 4 | 18.9 | (15.4-22.4) |  | 17.6 | (14.2-20.9) |  | 16.0 | (12.8-19.2) |  | 16.3 | (13-19.5) |  | 16.4 | (13-19.7) |  | 14.7 | (11.5-17.9) |
| Most deprived | 18.8 | (15.2-22.4) |  | 18.7 | (15.2-22.2) |  | 20.8 | (17.2-24.5) |  | 18.2 | (14.8-21.6) |  | 16.4 | (13-19.7) |  | 20.1 | (16.4-23.8) |
| Overall | 15.6 | (14.2-17) |  | 16.0 | (14.6-17.4) |  | 14.0 | (12.7-15.3) |  | 13.9 | (12.6-15.3) |  | 13.3 | (12-14.6) |  | 13.6 | (12.2-15) |
|  |  |  |  |  |  |  |  |  |  |  |  |  |  |  |  |  |  |
| SII | 8.8 | (4-13.7) |  | 6.4 | (1.3-11.6) |  | 14.4 | (10.1-18.5) |  | 11.5 | (7.2-15.9) |  | 8.8 | (4.3-13.4) |  | 10.6 | (5.5-15.4) |
| RII_L_ | 0.05 | (0.03-0.08) |  | 0.04 | (0.01-0.07) |  | 0.09 | (0.07-0.11) |  | 0.08 | (0.05-0.11) |  | 0.07 | (0.03-0.1) |  | 0.07 | (0.04-0.1) |
| % from total SII | 2.9 |  |  | 2.3 |  |  | 5.0 |  |  | 4.4 |  |  | 3.7 |  |  | 3.8 |  |
